# 3-Chloro-*N*′-[(2-hy­droxy­naphthalen-1-yl)methyl­idene]benzohydrazide

**DOI:** 10.1107/S1600536811000912

**Published:** 2011-01-12

**Authors:** Tian-Yi Li, Wei Li

**Affiliations:** a School of Chemical Engineering, Changchun University of Technology, Changchun 130012, People’s Republic of China

## Abstract

The title compound, C_18_H_13_ClN_2_O_2_, was prepared by the reaction of 2-hy­droxy-1-naphthaldehyde with 3-chloro­benzohydrazide in methanol. An intra­molecular O—H⋯N hydrogen bond influences the mol­ecular conformation; the benzene ring and naphthyl ring system form a dihedral angle of 17.1 (3)°. In the crystal, inter­molecular N—H⋯O hydrogen bonds link the mol­ecules into chains propagated in [101].

## Related literature

For Schiff base compounds, see: Bessy *et al.* (2006 ▶); Podyachev *et al.* (2007 ▶); Raj & Kurup (2007 ▶); Pouralimardan *et al.* (2007 ▶); Bacchi *et al.* (2006 ▶); Dinda *et al.* (2002 ▶). For reference bond lengths, see: Allen *et al.* (1987 ▶). For details of the synthesis, see: Zhu (2010 ▶).
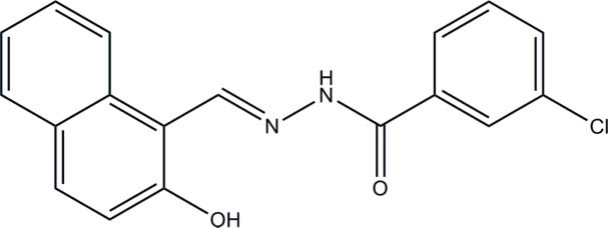


## Experimental

### 

#### Crystal data

C_18_H_13_ClN_2_O_2_
*M* *_r_* = 324.75
Monoclinic, 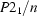

*a* = 7.158 (2) Å
*b* = 30.886 (3) Å
*c* = 7.3733 (12) Å
β = 108.924 (2)°
*V* = 1541.9 (5) Å^3^
*Z* = 4
Mo *K*α radiation
μ = 0.26 mm^−1^
*T* = 298 K
0.20 × 0.20 × 0.18 mm

#### Data collection

Bruker APEXII CCD area-detector diffractometer
Absorption correction: multi-scan (*SADABS*; Bruker, 2005 ▶) *T*_min_ = 0.950, *T*_max_ = 0.955
8306 measured reflections
3288 independent reflections
1635 reflections with *I* > 2σ(*I*)
*R*_int_ = 0.062

#### Refinement

*R*[*F* ^2^ > 2σ(*F* ^2^)] = 0.082
*wR*(*F* ^2^) = 0.161
*S* = 1.04
3288 reflections
212 parameters
1 restraint
H atoms treated by a mixture of independent and constrained refinement
Δρ_max_ = 0.24 e Å^−3^
Δρ_min_ = −0.35 e Å^−3^

### 

Data collection: *APEX2* (Bruker, 2005 ▶); cell refinement: *SAINT* (Bruker, 2005 ▶); data reduction: *SAINT*; program(s) used to solve structure: *SHELXTL* (Sheldrick, 2008 ▶); program(s) used to refine structure: *SHELXTL*; molecular graphics: *SHELXTL*; software used to prepare material for publication: *SHELXTL*.

## Supplementary Material

Crystal structure: contains datablocks global, I. DOI: 10.1107/S1600536811000912/cv5033sup1.cif

Structure factors: contains datablocks I. DOI: 10.1107/S1600536811000912/cv5033Isup2.hkl

Additional supplementary materials:  crystallographic information; 3D view; checkCIF report

## Figures and Tables

**Table 1:** Hydrogen-bond geometry (Å, °)

*D*—H⋯*A*	*D*—H	H⋯*A*	*D*⋯*A*	*D*—H⋯*A*
N2—H2⋯O2^i^	0.90 (3)	1.99 (2)	2.860 (4)	162 (4)
O1—H1⋯N1	0.82	1.85	2.574 (4)	146

Symmetry code: (i) 
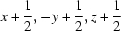
.

**Fig. 1.**
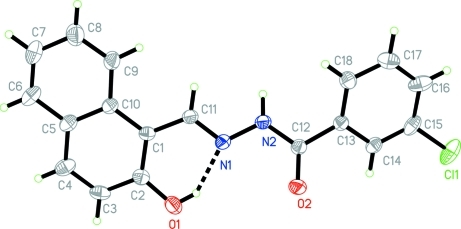
The molecular structure of the title compound with 30% probability displacement ellipsoids for non-hydrogen atoms. Intramolecular O—H···N hydrogen bond is drawn as a dashed line.

**Fig. 2.**
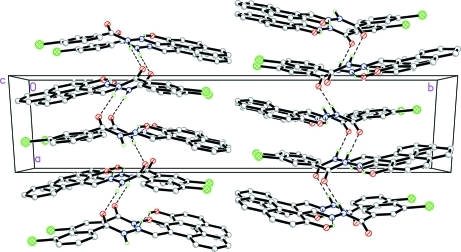
A portion of the crystal packing showing H-bonds as dashed lines.

## supplementary crystallographic information

### Comment

In the last years, a number of Schiff bases derived from the reaction of aldehydes with benzohydrazides were prepared and structurally characterized (Bessy *et al.*, 2006; Podyachev *et al.*, 2007; Raj & Kurup, 2007; Pouralimardan *et al.*, 2007; Bacchi *et al.*, 2006; Dinda *et al.*, 2002). As a contribution to this work, we present here the title new Schiff base compound (Fig. 1).

There is an intramolecular O—H···N hydrogen bond in the molecule, which influences the molecular conformation - the dihedral angle between the benzene ring and the naphthyl ring is 17.1 (3)°. All the bond lengths are within normal values (Allen *et al.*, 1987). In the crystal structure, intermolecular N—H···O hydrogen bonds (Table 1) link the molecules into chains propagated in [101] (Fig. 2).

### Experimental

The compound was prepared and crystallized according to the literature method (Zhu, 2010). 2-Hydroxy-1-naphthaldehyde (0.172 g, 1 mmol) and 3-chlorobenzohydrazide (0.171 g, 1 mmol) were dissolved in 30 ml absolute methanol. The mixture was stirred at reflux for 10 min, and cooled to room temperature. The clear colorless solution was left to slow evaporation in air for five days, yielding colorless block-shaped crystals, which were collected by filtration and washed with methanol.

### Refinement

The amino H atom was located from a difference Fourier map and refined isotropically, with the N—H distance restrained to 0.90 (1) Å. The other H atoms were positioned geometrically and refined using the riding-model approximation, with C—H = 0.93 Å, and O—H = 0.82 Å, and *U*_iso_(H) = 1.2*U*_eq_(C) or *U*_iso_(H) = 1.5*U*_eq_(O).

### Crystal data

**Table:**

C_18_H_13_ClN_2_O_2_	*F*(000) = 672
*M**_r_* = 324.75	*D*_x_ = 1.399 Mg m^−^^3^
Monoclinic, *P*2_1_/*n*	Mo *K*α radiation, λ = 0.71073 Å
*a* = 7.158 (2) Å	Cell parameters from 1332 reflections
*b* = 30.886 (3) Å	θ = 2.5–24.9°
*c* = 7.3733 (12) Å	µ = 0.26 mm^−^^1^
β = 108.924 (2)°	*T* = 298 K
*V* = 1541.9 (5) Å^3^	Block, colorless
*Z* = 4	0.20 × 0.20 × 0.18 mm

### Data collection

**Table:**

Bruker APEXII CCD area-detector diffractometer	3288 independent reflections
Radiation source: fine-focus sealed tube	1635 reflections with *I* > 2σ(*I*)
graphite	*R*_int_ = 0.062
ω scans	θ_max_ = 27.0°, θ_min_ = 2.6°
Absorption correction: multi-scan (*SADABS*; Bruker, 2005)	*h* = −9→9
*T*_min_ = 0.950, *T*_max_ = 0.955	*k* = −32→39
8306 measured reflections	*l* = −9→6

### Refinement

**Table:**

Refinement on *F*^2^	Primary atom site location: structure-invariant direct methods
Least-squares matrix: full	Secondary atom site location: difference Fourier map
*R*[*F*^2^ > 2σ(*F*^2^)] = 0.082	Hydrogen site location: inferred from neighbouring sites
*wR*(*F*^2^) = 0.161	H atoms treated by a mixture of independent and constrained refinement
*S* = 1.04	*w* = 1/[σ^2^(*F*_o_^2^) + (0.0443*P*)^2^ + 0.9339*P*] where *P* = (*F*_o_^2^ + 2*F*_c_^2^)/3
3288 reflections	(Δ/σ)_max_ < 0.001
212 parameters	Δρ_max_ = 0.24 e Å^−^^3^
1 restraint	Δρ_min_ = −0.35 e Å^−^^3^

### Special details

**Table:**

Geometry. All e.s.d.'s (except the e.s.d. in the dihedral angle between two l.s. planes) are estimated using the full covariance matrix. The cell e.s.d.'s are taken into account individually in the estimation of e.s.d.'s in distances, angles and torsion angles; correlations between e.s.d.'s in cell parameters are only used when they are defined by crystal symmetry. An approximate (isotropic) treatment of cell e.s.d.'s is used for estimating e.s.d.'s involving l.s. planes.
Refinement. Refinement of *F*^2^ against ALL reflections. The weighted *R*-factor *wR* and goodness of fit *S* are based on *F*^2^, conventional *R*-factors *R* are based on *F*, with *F* set to zero for negative *F*^2^. The threshold expression of *F*^2^ > σ(*F*^2^) is used only for calculating *R*-factors(gt) *etc*. and is not relevant to the choice of reflections for refinement. *R*-factors based on *F*^2^ are statistically about twice as large as those based on *F*, and *R*- factors based on ALL data will be even larger.

### Fractional atomic coordinates and isotropic or equivalent isotropic displacement parameters (Å^2^)

**Table:**

	*x*	*y*	*z*	*U*_iso_*/*U*_eq_	
Cl1	0.66632 (19)	0.05960 (4)	0.6685 (2)	0.0843 (5)	
H2	0.696 (5)	0.2651 (13)	0.585 (3)	0.080*	
N1	0.5796 (4)	0.28518 (9)	0.3225 (4)	0.0418 (8)	
N2	0.6082 (5)	0.25640 (10)	0.4729 (4)	0.0427 (8)	
O1	0.5149 (5)	0.30417 (9)	−0.0326 (4)	0.0597 (8)	
H1	0.5222	0.2884	0.0590	0.090*	
O2	0.4224 (4)	0.20537 (8)	0.2758 (4)	0.0533 (8)	
C1	0.6241 (5)	0.35642 (11)	0.2236 (5)	0.0360 (9)	
C2	0.5645 (5)	0.34493 (13)	0.0298 (6)	0.0445 (10)	
C3	0.5567 (6)	0.37613 (15)	−0.1110 (6)	0.0562 (12)	
H3	0.5214	0.3678	−0.2389	0.067*	
C4	0.5998 (6)	0.41809 (15)	−0.0627 (7)	0.0586 (12)	
H4	0.5922	0.4383	−0.1585	0.070*	
C5	0.6560 (5)	0.43187 (12)	0.1296 (6)	0.0461 (10)	
C6	0.6976 (6)	0.47585 (14)	0.1805 (7)	0.0609 (12)	
H6	0.6873	0.4962	0.0848	0.073*	
C7	0.7521 (7)	0.48893 (14)	0.3655 (8)	0.0706 (14)	
H7	0.7803	0.5179	0.3966	0.085*	
C8	0.7655 (7)	0.45872 (15)	0.5081 (7)	0.0716 (14)	
H8	0.8008	0.4677	0.6351	0.086*	
C9	0.7276 (6)	0.41613 (13)	0.4649 (6)	0.0545 (11)	
H9	0.7406	0.3965	0.5640	0.065*	
C10	0.6693 (5)	0.40069 (12)	0.2746 (6)	0.0398 (9)	
C11	0.6392 (5)	0.32393 (12)	0.3679 (5)	0.0397 (9)	
H11	0.6933	0.3313	0.4967	0.048*	
C12	0.5277 (5)	0.21672 (12)	0.4356 (5)	0.0401 (9)	
C13	0.5780 (5)	0.18679 (11)	0.6038 (5)	0.0375 (9)	
C14	0.5923 (5)	0.14310 (12)	0.5659 (6)	0.0426 (9)	
H14	0.5690	0.1337	0.4407	0.051*	
C15	0.6411 (5)	0.11390 (12)	0.7143 (7)	0.0507 (11)	
C16	0.6694 (6)	0.12736 (16)	0.8992 (7)	0.0620 (13)	
H16	0.7013	0.1074	0.9989	0.074*	
C17	0.6502 (6)	0.17043 (16)	0.9362 (6)	0.0609 (12)	
H17	0.6675	0.1795	1.0609	0.073*	
C18	0.6053 (5)	0.20038 (13)	0.7888 (6)	0.0476 (10)	
H18	0.5936	0.2296	0.8143	0.057*	

### Atomic displacement parameters (Å^2^)

**Table:**

	*U*^11^	*U*^22^	*U*^33^	*U*^12^	*U*^13^	*U*^23^
Cl1	0.0781 (9)	0.0429 (7)	0.1416 (13)	0.0095 (6)	0.0490 (8)	0.0229 (7)
N1	0.0476 (19)	0.0348 (18)	0.040 (2)	−0.0005 (14)	0.0096 (15)	0.0034 (15)
N2	0.047 (2)	0.0355 (18)	0.0368 (19)	−0.0048 (15)	0.0017 (15)	0.0038 (16)
O1	0.078 (2)	0.0557 (19)	0.0442 (18)	−0.0086 (16)	0.0175 (16)	−0.0077 (14)
O2	0.0568 (17)	0.0465 (16)	0.0417 (17)	−0.0031 (13)	−0.0047 (13)	−0.0018 (13)
C1	0.0287 (19)	0.044 (2)	0.035 (2)	0.0038 (16)	0.0104 (16)	0.0037 (18)
C2	0.043 (2)	0.051 (3)	0.042 (2)	0.0003 (19)	0.0166 (19)	0.001 (2)
C3	0.062 (3)	0.074 (3)	0.032 (2)	0.003 (2)	0.015 (2)	0.011 (2)
C4	0.062 (3)	0.059 (3)	0.062 (3)	0.005 (2)	0.029 (2)	0.021 (2)
C5	0.040 (2)	0.044 (3)	0.057 (3)	0.0054 (18)	0.019 (2)	0.009 (2)
C6	0.059 (3)	0.044 (3)	0.082 (4)	0.005 (2)	0.026 (3)	0.020 (3)
C7	0.077 (3)	0.041 (3)	0.099 (4)	−0.007 (2)	0.035 (3)	−0.001 (3)
C8	0.089 (4)	0.058 (3)	0.072 (4)	−0.015 (3)	0.032 (3)	−0.011 (3)
C9	0.068 (3)	0.041 (3)	0.057 (3)	−0.008 (2)	0.025 (2)	−0.002 (2)
C10	0.034 (2)	0.041 (2)	0.045 (2)	0.0035 (16)	0.0134 (18)	0.004 (2)
C11	0.040 (2)	0.040 (2)	0.036 (2)	0.0024 (17)	0.0084 (17)	0.0051 (18)
C12	0.035 (2)	0.039 (2)	0.042 (2)	0.0006 (17)	0.0066 (18)	−0.0004 (19)
C13	0.035 (2)	0.036 (2)	0.039 (2)	−0.0010 (16)	0.0091 (17)	0.0009 (18)
C14	0.038 (2)	0.037 (2)	0.052 (3)	−0.0011 (17)	0.0130 (18)	0.006 (2)
C15	0.036 (2)	0.035 (2)	0.083 (4)	0.0019 (17)	0.021 (2)	0.011 (2)
C16	0.046 (3)	0.073 (3)	0.064 (3)	−0.002 (2)	0.014 (2)	0.031 (3)
C17	0.061 (3)	0.075 (3)	0.047 (3)	−0.006 (2)	0.018 (2)	0.010 (3)
C18	0.046 (2)	0.049 (2)	0.048 (3)	−0.0023 (19)	0.016 (2)	−0.003 (2)

### Geometric parameters (Å, °)

**Table:**

Cl1—C15	1.732 (4)	C6—H6	0.9300
N1—C11	1.278 (4)	C7—C8	1.386 (6)
N1—N2	1.384 (4)	C7—H7	0.9300
N2—C12	1.344 (4)	C8—C9	1.360 (5)
N2—H2	0.90 (3)	C8—H8	0.9300
O1—C2	1.348 (4)	C9—C10	1.410 (5)
O1—H1	0.8200	C9—H9	0.9300
O2—C12	1.226 (4)	C11—H11	0.9300
C1—C2	1.398 (5)	C12—C13	1.494 (5)
C1—C10	1.427 (5)	C13—C18	1.379 (5)
C1—C11	1.441 (5)	C13—C14	1.388 (5)
C2—C3	1.404 (5)	C14—C15	1.373 (5)
C3—C4	1.353 (5)	C14—H14	0.9300
C3—H3	0.9300	C15—C16	1.376 (6)
C4—C5	1.408 (5)	C16—C17	1.374 (6)
C4—H4	0.9300	C16—H16	0.9300
C5—C6	1.415 (5)	C17—C18	1.383 (5)
C5—C10	1.419 (5)	C17—H17	0.9300
C6—C7	1.353 (6)	C18—H18	0.9300
			
C11—N1—N2	116.2 (3)	C8—C9—H9	118.9
C12—N2—N1	118.6 (3)	C10—C9—H9	118.9
C12—N2—H2	126 (3)	C9—C10—C5	116.5 (4)
N1—N2—H2	115 (3)	C9—C10—C1	123.7 (3)
C2—O1—H1	109.5	C5—C10—C1	119.8 (4)
C2—C1—C10	118.7 (3)	N1—C11—C1	121.3 (3)
C2—C1—C11	120.2 (3)	N1—C11—H11	119.3
C10—C1—C11	121.1 (3)	C1—C11—H11	119.3
O1—C2—C1	123.1 (3)	O2—C12—N2	123.1 (3)
O1—C2—C3	116.5 (4)	O2—C12—C13	121.9 (3)
C1—C2—C3	120.5 (4)	N2—C12—C13	115.0 (3)
C4—C3—C2	120.8 (4)	C18—C13—C14	119.9 (3)
C4—C3—H3	119.6	C18—C13—C12	123.4 (3)
C2—C3—H3	119.6	C14—C13—C12	116.6 (3)
C3—C4—C5	121.4 (4)	C15—C14—C13	119.6 (4)
C3—C4—H4	119.3	C15—C14—H14	120.2
C5—C4—H4	119.3	C13—C14—H14	120.2
C4—C5—C6	121.6 (4)	C14—C15—C16	120.6 (4)
C4—C5—C10	118.8 (4)	C14—C15—Cl1	119.8 (4)
C6—C5—C10	119.6 (4)	C16—C15—Cl1	119.6 (3)
C7—C6—C5	121.4 (4)	C17—C16—C15	119.8 (4)
C7—C6—H6	119.3	C17—C16—H16	120.1
C5—C6—H6	119.3	C15—C16—H16	120.1
C6—C7—C8	119.4 (4)	C16—C17—C18	120.3 (4)
C6—C7—H7	120.3	C16—C17—H17	119.9
C8—C7—H7	120.3	C18—C17—H17	119.9
C9—C8—C7	120.9 (5)	C13—C18—C17	119.7 (4)
C9—C8—H8	119.5	C13—C18—H18	120.1
C7—C8—H8	119.5	C17—C18—H18	120.1
C8—C9—C10	122.1 (4)		

### Hydrogen-bond geometry (Å, °)

**Table:**

*D*—H···*A*	*D*—H	H···*A*	*D*···*A*	*D*—H···*A*
N2—H2···O2^i^	0.90 (3)	1.99 (2)	2.860 (4)	162 (4)
O1—H1···N1	0.82	1.85	2.574 (4)	146

Symmetry codes: (i) *x*+1/2, −*y*+1/2, *z*+1/2.
